# Extended Skill Learning

**DOI:** 10.3389/fpsyg.2020.01956

**Published:** 2020-08-14

**Authors:** Edward Baggs, Vicente Raja, Michael L. Anderson

**Affiliations:** ^1^Rotman Institute of Philosophy, University of Western Ontario, London, ON, Canada; ^2^Department of Philosophy, University of Western Ontario, London, ON, Canada; ^3^Brain and Mind Institute, University of Western Ontario, London, ON, Canada

**Keywords:** skill learning, embodied cognition, ecological psychology, enactivism, animal–environment system, psychological explanation

## Abstract

Within the ecological and enactive approaches in cognitive science, a tension exists in how the process of skill learning is understood. Skill learning can be understood in a narrow sense, as a process of bodily change over time, or in an extended sense, as a change in the structure of the animal–environment system. We propose to resolve this tension by rejecting the first understanding in favor of the second. We thus defend an extended approach to skill learning. An extended understanding of skill learning views bodily changes as being embedded in a larger process of interaction between the organism and specific structures in the environment. Such an extended approach is committed to the claims that (1) the appropriate unit of analysis for understanding skill learning is not the body but the activity and (2) learning consists in the establishment and adaptive organization of enabling constraints on that activity. We focus on two example cases: maintaining upright posture and walking. In both cases, environmental structures play a constitutive role in the activity throughout learning, but the specific environmental structures that are involved in the activity change over time. At an early stage, the child makes use of an environmental “support”—for example, holding onto furniture to maintain upright posture. Later, once further constraints have been established, the child is able to let go of the furniture and remain upright. We argue that adopting an extended understanding of skill learning offers a promising strategy for unifying ecological and enactive approaches and can also potentially ground a radically embodied approach to higher cognition.

## Introduction: Two Senses of Skill

One promising potential area of convergence between the ecological and enactive approaches in cognitive science is in the development of a general theory of skill learning. Theoretical work within both approaches has come increasingly, in recent years, to appeal to the notion of skill as an explanatory factor in the understanding of behavior (e.g., Chemero, 2009; Rietveld and Kiverstein, 2014; Di Paolo et al., 2017; Baggs and Chemero, 2020). This is particularly true in the case of attempts to explain specifically human forms of behavior, namely those involving language. To be a competent well-adjusted adult human, so the story goes, is to exercise a set of skills in an appropriate way in a variety of contexts.

Within current theoretical writing within these approaches, however, a tension seems to exist in how the notion of “skill” is used. On the one hand, the word is used as if it denotes some property of the animal’s body. The body is said to “possess” a set of skills or to be constituted as a network of such skills. On the other hand, the word is used to denote the performance of some activity. When “skill” is invoked in this second sense, it seems that the concept can no longer be understood as referring narrowly to some property of the body, but must be understood as an extended phenomenon spanning the animal–environment system.

Two examples will suffice to illustrate this tension. First, Rietveld and Kiverstein (2014, p. 325, emphasis added), in their work that seeks to expand the theoretical scope of ecological psychology, tell us that “the affordances an environment offers to an animal are dependent on the skills the animal *possesses*”. On the face of it, this is an instance of treating skills as a property of the body (a possession). But it is clear that these authors do not want to view a skill as simply a property of the organism. Elsewhere in the paper, the authors endorse the claim of Gibson (1979) that learning involves the “education of attention.” They write (Rietveld and Kiverstein, 2014, p. 331): “In acquiring a skill, we learn in which places in the environment to find the affordances relevant to our concerns and what aspects of [the] environment to attend to.” This seems to imply, in contrast, that the learning and exercising of a skill *inherently* involves the environment: skillful acting simply is directing one’s attention to something *in* the environment. In that case, it is misleading to say that the skill is something that can straightforwardly be considered a property of the organism’s body alone.

Second, Di Paolo et al. (2017, p. 196), in developing their enactivist account, propose that “a cognitive agent” (an animal) can be conceived “as essentially an integrated ecology of sensorimotor skills.” Building on Piaget, these authors take skill learning to involve the construction and progressive elaboration of a network of sensorimotor schemes. The suggestion is that we somehow “incorporate” these schemes into our bodies. This appears, once again, to be an instance of skill-as-bodily-property thinking. Yet elsewhere, these authors insist that skills are in fact something other than simply properties that the organism’s body possesses. Skill learning is said to be “world-involving.” Skill learning leads to mastery, which “is a world-involving concept since it relies on dynamic engagements with the world, enacted or potential” (Di Paolo et al., 2017, p. 107). Again, it seems that skill-as-bodily-property thinking is too restrictive to capture the theory of skill learning that these authors are actually trying to develop. The tension that we have identified seems to run through both of these contributions.

How might we resolve this tension? A standard formulation proposes that learning should be understood not in terms of the accumulation of bodily properties but in terms of change. Instead of viewing the learner as gathering more and more “knowledge” of its environment, we should view the learner as changing so as to become increasingly adapted to the structure of that environment (e.g., Gibson and Gibson, 1955; Pacheco et al., 2019). Similarly, Araújo and Davids (2011) suggest that it is a mistake to use the phrase “skill acquisition” to refer to this process. To frame the investigation in terms of “acquisition” is already to seek an explanation of learning in terms of an accumulation of bodily properties. Araújo and Davids (2011) suggest that we should abandon talk of “skill acquisition” in favor of terms such as “skill adaptation” or “skill attunement.” We agree with this. It is important to note, however, that what Araújo and Davids are in fact advocating for here is not merely a change of wording, but a change in the scale of analysis at which we understand what skills are in the first place.

The key to resolving the tension, then, is to appreciate that the two senses of skill—skills as bodily properties vs. as properties of the extended organism–environment system—are simply two ways of describing the outcome of a single process. Specifically, the two senses of “skill” are describing the same process of learning at two different scales of analysis—namely, the bodily scale and the ecological scale. In practice, it only really makes sense to talk of skills at the scale of the activity, not at the scale of the body. Of course, the body does change over the course of learning, and this change includes changes in the nervous system. But, crucially, those changes do not arise autonomously within the body alone (and thus it is odd to say that the body “possesses” the skill). Rather, skills arise always *through situated engagement with an environment*. Bodily change should, therefore, be understood as bodily-change-relative-to-an-environment or, even better, as a change in the extended structure of the animal–environment system. In short, skill learning is an inherently extended phenomenon.

In what follows, we will be drawing on work from the empirical literature on motor control in infancy and in later learning. We highlight this work in order to illustrate the claim that skill learning, in practice, can only ever be understood as an extended phenomenon that constitutively involves structure not just in the animal’s body but also in the animal’s environment.

Toward the end of the paper, we will turn to the question of how adopting this extended view of skill learning might help to unify the ecological and enactive approaches. Generally speaking, we envision a future ecological–enactive account of skills, which recognizes that (i) the appropriate unit of analysis for understanding skill learning is not the body itself, but the activity that spans organism and environment and (ii) learning consists in the establishment and the adaptive organization of *enabling constraints* on that activity (Anderson, 2015; Raja and Anderson, 2020). This view allows for a flexible and general account of skill learning, one that is equally appropriate for describing learning in motor tasks such as learning to walk, and learning in social situations, which should be understood in terms of action relative to an environment that is populated with other actors. First, we consider some examples from infant motor control.

## Two Examples of Skill Learning

### Upright Posture

Learning to maintain an upright posture is an important developmental milestone in typically developing children. Children generally learn to stand unaided sometime around their first birthday, though it takes years of learning for an individual to be able to maintain the upright posture in a wide variety of different contexts and situations (Adolph, 2008). Some of the factors necessary for upright posture are seemingly straightforwardly features of the body. The first requirement is that the infant develops sufficient bodily strength for overcoming gravity (McGraw, 1932; notice that already here we are referring to gravity, i.e., a feature of the animal–environment system). Other requirements include anatomical and biomechanical changes, such as in the spine and the pelvis (Lovejoy, 2005a), the hip and thigh (Lovejoy, 2005b), or the knee (Lovejoy, 2007).

Maintaining the upright posture is also a perceptual task. In simple terms, it calls for continuous compensatory movements to control the position and momentum of the center of gravity of the body in order to hold it within the limits of its base of support—that is, the area of contact between the body and the supporting surface in the environment (Riley et al., 1995; Krebs et al., 2002).

An illustrative example of the role of visual perception in maintaining the upright posture is the moving room experiment (Lee and Aronson, 1974; Lee and Lishman, 1975). In this experiment, participants stand within a room that looks completely normal. In fact, however, the walls of the room are mounted on rails (or else the room is suspended on ropes from above) so experimenters can “move the room” relative to the participant. A participant may therefore be standing still looking at a wall and nevertheless she may see the wall approaching or receding away from her. The movement of the walls generates different patterns of optic flow (i.e., changes in the visual field of the participant) that directly affect the participant’s capacity to maintain upright posture. The flow generated by an approaching wall, also known as optic push, often causes the participant to lose her balance. The effect is especially dramatic when the participant is a toddler. The approaching wall can easily cause the child to lose control of the upright posture and fall to the floor (Lee and Aronson, 1974). The same optic push does not affect adults to quite the same extent. As long as the adult participant is standing on a wide enough base of support, for example, she is standing on a regular floor, the participant will not typically stumble or fall over. But if the base of support is thinner or less stable than usual, for example, if the participant is asked to stand on a narrow wooden beam, the adult participant will typically have more difficulty staying upright and may be forced to make strong corrective movements or to step off the beam (Lee and Lishman, 1975; see also the differences in standing on the land or on a ship at sea in Stoffregen et al., 2011).

For present purposes, we are interested in the question of how it is that children learn to stand upright in the first place. It is noteworthy that at its earliest stages, the task involves environmental support in a very immediate way. Infants, before they learn to stand unaided, typically first pull themselves up on furniture and other object, maintaining upright posture by leaning on the object with their hands or torso. It is commonly said that this behavior allows the infant to “augment” her balance (e.g., Berger et al., 2013). This is surely true. But another way to describe this pulling-to-stand activity is to say that the furniture item is in fact itself part of the learning process that is necessary in order to reach a mature task solution. That is, postural control extends from the body of the learner to incorporate the solid structures she encounters in her environment.

By holding onto the furniture, the child has achieved temporary postural stability. The child’s postural degrees of freedom have been frozen, in a sense. But notice that this is not achieved simply by freezing the degrees of freedom internal to the child’s musculoskeletal system. In effect, the furniture item has been incorporated into the postural control system, and it is this that provides temporary stability (we will discuss the degrees of freedom problem in more detail in the next section).

Holding onto the furniture, the child is now free to explore her motor space in a new way. She is free to explore the kinds of perceptual information that are generated when she arranges her lower limbs into the arrangement necessary for standing. Crucially, this kind of information (visual information about the room, haptic information about the angles of the joints, the weight of the body on the limbs, etc.) can *only* be explored by actually adopting an upright standing position. As long as the child holds onto the furniture, the solution space is constrained and some of the degrees of freedom of the system have been fixed (e.g., having pulled herself up on the furniture, the child cannot move from *here*, except by lowering herself again). Later, once the child has sufficiently explored this new motor solution space, she learns to control her posture relative to some structure in this new information field. Eventually, the child is able to let go of the furniture. At this point, the postural task has become different in nature, but note that it remains equally extended into the environment. Now, instead of relying on a single item of furniture to stay where it is, the child relies on the global layout of the whole environment to stay roughly where it is—we might say that the motor constraint offered by the furniture is replaced by a set of perceptual constraints enabled by the optic flow, the gravitational vector, and so on.

### Walking

As we have just seen, maintaining an upright posture involves swaying so as to *cancel out* optic flow relative to the environment (assuming the environment is stable and you are not standing in a moving room). Walking and locomotion in general is different. Walking involves *generating* optic flow in a more or less continuous manner, in order to control movement in a desired direction. Optic flow, in the case of locomotion, is the information that specifies whether or not the actor is successfully moving from “here” to “over there.”

But again, optic flow alone is not enough. A number of other enabling factors are required in order for walking to occur. These include postural stability, sufficiently strong muscles and bones, a motivation to move in a particular direction, and an appropriate surface of support (Thelen and Smith 1994, p. 20; see also Adolph et al., 2012).

A classic illustration of one of the relevant constraints at play is provided by Thelen’s work on the spontaneous “stepping” motion in infants (Thelen, 1984; Thelen and Smith, 1994). Newborn infants, when held upright, will often spontaneously exhibit a pattern of leg movement that looks like stepping—that is, the infant will exhibit alternate rhythmic movements of the left and right legs (McGraw, 1932). This behavior, however, “disappears” at around 2 months. Typically, the stepping pattern does not “reappear” until the child begins to walk by herself toward the end of the first year. This “U-shaped” developmental pattern had long been a puzzle in infant movement research. When Thelen and her colleagues investigated this, they discovered that the stepping behavior could be re-induced by various methods. For instance, a 3-month-old infant might show the stepping pattern if held in water, rather than over the ground. Or a 7-month-old infant might show a natural-looking stepping gait when held over a treadmill, rather than a stationary substrate (Thelen and Smith, 1994). So what is going on here? On the face of it, it seems as though the child does not actually lose the *ability* to produce the rhythmic pattern, but simply stops doing it, for some reason. Thelen and her colleagues were able to offer a persuasive explanation. The reason for the “disappearance” of the stepping pattern in the first months, they argued, is that infants quite rapidly gain body weight in this early period and the weight gain occurs faster than the gain in leg muscle. For 2-month-old infants, the problem is simply that their legs have gotten too fat for it to be worth assembling the stepping pattern (this explanation is supported by a wealth of evidence; for details, see Thelen and Smith, 1994, chapter 4).

Notice that on Thelen’s interpretation, it is not the case that stepping is a “skill” that the infant can be straightforwardly said to alternately possess, and then not possess, and then possess once more. It would make little sense to say that newborn infants “possess a stepping skill,” which they then “lose,” only to “reacquire” the same skill later in the year. Thelen herself understood this developmental phenomenon as a demonstration that causal explanations of infant development cannot appeal only to a single cause, such as the presence of some structure in the central nervous system, but must appeal instead to the whole situation supporting the activity. Causation is spread across body and environment: “There is … *no essence* of locomotion either in the motor cortex or in the spinal cord. Indeed, it would be equally credible to assign the essence of walking to the treadmill than to the neural structure, because it is the action of the treadmill that elicits the most locomotor-like behavior” (Thelen and Smith, 1994, p. 17, emphasis in original). Notice that Thelen and Smith are here already offering what we are calling an *extended* account of learning to walk.

We noted above that, at its earliest stages, maintaining upright posture constitutively involves the environment: infants pull themselves up to stand against furniture. The same is true of learning to walk. Characteristically, early walking is supported in some way by structure external to the infant’s body—either by furniture items, which the infant holds onto while shuffling, “cruising,” along (e.g., Haehl et al., 2000; Berger et al., 2013), by an adult holding onto the infant’s torso or hands as the infant is allowed to move her feet (McGraw, 1932), or by some specially constructed device such as a baby walker with wheels.

Again, these external “supports” can be thought of in a particular way: not merely as background conditions but as constitutive or necessary constraints on the infant’s activity and on the process of skill learning. Just as it is not possible to learn to stand except by adopting the standing posture (by, say, holding onto furniture to gain better control over the degrees of freedom relevant for the task), so it is not possible to learn to walk except by alternately planting your feet on the ground and moving forward, thus generating the relevant information about bodily posture—joint angles, momentum, vestibular flow, and so on. The infant’s activity, at this early “supported” stage of walking, is constrained in the sense that her body is temporarily coupled to another object or person. She cannot move around in this way except by, say, holding on to the fingers of a parent. As soon as she lets go of the fingers she slumps to the floor. In other words, the presence of the constraint (holding onto the parent) is a necessary condition for assembling the relevant motor solution. The learning of the skill is therefore an extended animal–environment event. Later on, after extensive practice in this “supported” manner of walking, the infant will let go of the fingers and begin to take her first steps “unaided.” When this occurs, the infant is demonstrating that she has gained some mastery over her internal postural control during walking and she no longer needs the postural constraint provided from outside her body. She has freed herself from one concrete externally-provided constraint and is now free to explore the motor space of this new walking posture relative to a moving pattern of optic flow (which, once again, is still an environmental constraint). She is free to explore her surroundings.

## Skill Learning as the Establishing of Enabling Constraints

We have discussed two simple examples of skill learning. These examples are sufficient to show that skill-as-bodily-property thinking is inadequate for capturing the process by which a skill is learned. As soon as we begin to look at the details of the learning process in a given case, it becomes apparent that we need to understand learning not merely in terms of bodily change, but also in terms of the environmental resources that are involved in the performance of the task. It is more useful, in fact, to think of learning as a process whereby a set of enabling constraints are established that allow the learner to carry out the task.

The concept of enabling constraint is a general concept that we have previously introduced in order to distinguish certain system-scale explanations from more reductionistic component-based explanations (Anderson 2015; Raja and Anderson, 2020). Roughly, an enabling constraint is something that limits the degrees of freedom of a system and thereby allows the system to perform some activity that would otherwise not be possible for the system. More formally, a constraint is a relationship between some system S and some set of entities or processes {X} such that {X} biases the probability of a set of possible outcomes/states for S. An *enabling* constraint is one that biases the set in favor of *positive, functional* outcomes for S (defined relative to S; Raja and Anderson, 2020). The concept is useful, for instance, for making sense of the activity of starburst amacrine cells in the mammalian retina: it is difficult to understand the direction-specific motion-detection function of the cell’s dendrites except by considering the cell as part of a larger system that constrains the activity of the cell to render it functional (Anderson, 2015). At a more macro scale, the concept of enabling constraint can also be applied to the behavior of the organism itself: the relatively slow movements of the organism, for instance, can be understood as constraining the relatively fast activity of the organism’s nervous system (Raja and Anderson, 2019; Raja, 2020; see also Van Orden et al., 2012).

We now suggest that the concept of enabling constraint can usefully be applied to understanding how skills are learned. Indeed, the concept of enabling constraint captures the way that skill learning is already understood by researchers working in several broadly embodied traditions who study the process of skill learning empirically. The notion of enabling constraint is consistent with at least the following three strands of current thinking in skill learning research.

First, consider Newell’s constraint-based theory of coordination (Newell, 1986; Pacheco et al., 2019). Newell (1986) distinguishes between three sources of constraint: organismic constrains, environmental constraints, and task constraints. Organismic constraints are such things as the strength of the infant’s limbs, mentioned above in relation to walking. Environmental constraints include such things as gravity, air temperature, lighting conditions, and also the medium in which the activity is carried out (for example, the infant’s stepping pattern can “re-emerge” when the infant is held in water; see Thelen, 1983). Task constraints include the task goal, the rules for carrying out the task correctly (for example, in race walking there is a task constraint that at least one of the participant’s feet must be in contact with the ground at all times), and the equipment used (a large soccer ball presents more difficulty to a small child than a smaller ball more appropriately scaled to the child’s body). The general notion of constraint is in fact ubiquitous in the literature on ecological and dynamic systems approaches to skill learning (see, e.g., Runeson, 1988; Vicente and Wang, 1998; Jacobs and Michaels, 2007; Davids et al., 2008). The way that constraints are invoked in this literature, including in Newell’s theory, can be understood in terms of enabling constraints at the scale of the task, that is, at the scale of the extended animal–environment interaction. On this constraints-based way of understanding things, it is inappropriate to say that a skill resides in the animal’s body alone (Araújo and Davids, 2011).

Second, it is often proposed that learning involves the freezing, followed by the freeing, of degrees of freedom in the motor system (Newell and van Emmerik, 1989; Vereijken et al., 1992; Guimarães et al., 2020). This process was originally proposed by Bernstein (1967), as a solution to a problem that he identified and that has come to be known as Bernstein’s problem: how does the motor system control a musculoskeletal system that seems to offer an arbitrarily large number of degrees of freedom? The proposal is that the motor system freezes some of the degrees of freedom in order to enable the assembling of a task solution. The process is characterized as involving three stages. In the first stage, the relevant degrees of freedom of the motor system for a given task are frozen out, meaning that they are kept rigid or fixed with respect to each other. In the second stage, individual degrees of freedom are de-frozen, allowing them to vary with respect to the other ones and progressively being integrated into functional units usually named *coordinative structures* (Kugler et al., 1980) or *synergies* (Kelso, 1995). Finally, the control strategy becomes more economical by exploiting passive forces (e.g., gravity or inertia) in the last stage of learning. In the case of the upright posture, one aspect of the learning process could for instance go from the freezing out of the joints in the legs to their combined control as a functional unit in which ankles, knees, and hips compensate each other and ending up in a better economy of balance by exploiting the inertial properties of the whole body (see Schneider et al., 1989, for an example of a similar process in the arms). Notice that coordinative structures or synergies are precisely instances of explanation in terms of enabling constraint.

This notion of freezing and freeing of degrees of freedom can be pushed further. Adopting an extended view of skill learning, we would say that in addition to recognizing the freezing of internal degrees of freedom *within the motor system*, we can also consider structures in the environment as providing constraints that enable the emergence of the activity. When the infant pulls to stand against a piece of furniture, she is freezing the degrees of freedom of her postural system relative to the furniture. By leaning on the furniture, the child is freezing the relevant degrees of freedom for the task in at least in two ways: in terms of the body, by making impossible some kinds of variations (e.g., rotating the forearm in the elbow–wrist axis) and in terms of the animal–environment system, also by making impossible some kinds of variation (e.g., moving too far away from the furniture such that her arm can no longer reach). In this sense, the environmental elements and the relation of the infant with them become an integral part of the learning of the skill: they are the way the process of mastering the control of degrees of freedom is extended beyond the body.

Third, the process of learning is often understood, within ecological and dynamical approaches, in terms of a search strategy (Pacheco et al., 2019). For instance, one of the main ecological theories of perceptual learning, *direct learning* (Jacobs and Michaels, 2007), understands learning as a change in the attunement to perceptual information, from not-so-good information to better information, to accomplish some task. Specifically, the learning process is understood as a search through the information space leading to a maximally optimal solution to the task. Learning to walk can be understood as a solution to the problem of locomoting through the environment (Adolph et al., 2012). The search for a solution is enabled by the infant’s establishing constraints on her own movements (holding on to furniture, etc.). The search of the information space leads the child to discover new enabling constraints. She discovers that it is possible for her to remain upright while keeping the rate of optic flow within some appropriately bounded region. She can let go of the furniture because new enabling constraints have been established that render the previous furniture-holding constraint no longer necessary.

The above considerations lead us to reject the concept of skill-as-bodily-property. It makes little sense to say that the child *acquires* a skill, or *possesses* it (again, we agree here with Araújo and Davids, 2011). Instead, it is more useful to understand skill learning as a re-organization of the entire extended system constituted by the actor, its environment, and the relational structure connecting the two. Skill learning is the establishing of enabling constraints at the scale of the task.

## Skillful Acting in a Populated Environment

The two main examples of skill learning that we have discussed so far are limited in various ways. Both are problems of motor control. In each case, movement is controlled relative to optic flow. And a similar set of constraints is involved in both cases (gravity, a suitable surface of support, muscle strength, etc.). More broadly, both are problems whose explanation can be conceived in terms of an *individual* actor encountering its own particular environmental surroundings. The examples are drawn from the literature on dynamical systems and motor development. Dynamic systems explanations have historically hewed to a version of methodological individualism (quite reasonably so, given the problem domains these approaches have been applied to). Explanation, in this approach, targets the system constituted by a single, individual actor and the relevant surroundings of that one actor. This is made explicit in certain places, such as in the following from Thelen and Smith (1994, p. 97): “A crucial assumption in a dynamic strategy is that *the individual and his or her behavioral changes over time are the fundamental unit of study*” (emphasis in original).

More recently, proponents of ecological and enactive approaches have sought to push explanation in cognitive science beyond the limitations imposed by methodological individualism (e.g., De Jaegher and Di Paolo, 2007; Schilbach et al., 2013; Chemero, 2016; Baggs et al., 2019). We think this rejection of methodological individualism is worth pursuing and we further suggest that the ideas outlined above are already general enough to be extended to social phenomena. An extended, relational account of skill learning offers a more powerful explanatory toolkit than has been suggested so far. Here, we will briefly consider two areas in which the extended view of skill learning may potentially be illuminating for social phenomena: in explaining the emergence of higher cognition and in explaining group activity.

Ultimately, theorists of radical embodiment seek to move beyond explanations of sensorimotor skills of the walking/standing upright type. We also want to be able to explain skills of the “higher”/symbolic type, such as language or counterfactual reasoning (see, e.g., Baggs, 2015; Sanches de Oliveira et al., 2019). The most promising framework for getting to the latter type of explanation remains that outlined by Vygotsky in the 1930s (Vygotsky, 1978). Vygotsky’s framework can be summarized quite succinctly. The basic story is the following. All actions start off as overt behavior. Counting to 10, for instance, initially consists precisely of speaking “out loud” the sequence of sounds “one, two, three…” This occurs, of course, in a social setting. A caregiver encourages the child to repeat the sequence and provides additional structure, for example, drawing the child’s attention to objects that are being “counted.” Over time, the child learns to coordinate the sequence of individual number words with attention to the sequence of individual objects. Eventually, the child is able to reliably produce the sequence of numbers in the appropriate order and to reliably coordinate the uttering of the individual number words along with the “counting” of individual objects. What was once a meaningless sequence of sounds has become a meaningful series of numbers and the child can now be said to have mastered, in some sense, the skill of counting. She can now engage in “higher” forms of social interaction that were previously impossible.

Note that the later forms of activity are not simply a more complicated version of the earlier form. At the earliest stage, the child is simply reproducing a sequence of sounds. Somehow, the child needs to discover that the individual numbers correspond to individual “countings” of objects. She needs to discover the relation, or the constraint, that connects the two structures. This discovery is facilitated by the actions of the caregiver. The caregiver “scaffolds” the discovery of the relation, to invoke the common metaphor (Wood et al., 1976). It should be noted that the child is always an active participant in this process. The caregiver acts so as to *constrain* the child’s utterances and to channel the child’s attention toward the objects. The outcome is that the initial task, reproducing a sound sequence, is transformed to a new activity, counting. But this new activity is still a world-directed activity. It is questionable whether it makes sense to say that anything has been “internalized” here. It is more accurate to say that the nature of the activity has changed and a new skill has emerged. A radical embodied account of language must begin with this kind of situated, embodied, attention-directing activity in early childhood (Reed, 1996; Baggs, 2015; Di Paolo et al., 2018; Van den Herik, 2018).

A constraint-based account of skill learning can also provide a valuable way to think about group activity. The world that we encounter in early childhood is a world that is *populated with other actors*. We live in a populated environment. A consequence of this is that other people (and animals) can constitute constraints on any given individual actor’s activity. We here wish to make explicit a claim that is latent in the discussion above. We have so far been appealing to the notion of enabling constraints as though such constraints only arise as an emergent consequence of the individual’s own behavior. But this is not the case. A baby walker, for instance, is an inanimate object that is encountered by the individual. But it is also a social object. It is designed specifically to assist learning, and it is provided to a child by a caregiver for that specific purpose.

A more radical claim could also be made here. We have proposed that skills should be understood as emergent properties of systems spanning animals and their environment. There is no reason in principle why we should not extend this and talk about skills at the scale of groups. In any team activity where there is a high degree of interdependence between the activities of the actors, such as in a soccer team, or between the staff on a hospital ward, the ability of any individual to achieve some desired outcome will be dependent on the skillful functioning of the system as a whole (Hutchins, 1995). In other words, structural properties of the team can influence, in a top-down fashion, the possibilities that are available to the individual members of the team. And, as discussed just above with reference to Vygotsky, such interdependent activity is characteristic of infant-caregiver interactions from early in life (Trevarthen and Aitken, 2001). So perhaps we should reject methodological individualism after all. Such a move—recognizing the primary role of interaction in skill learning—can potentially allow us to avoid the knotty set of issues that is encountered by theorists of social cognition who begin by assuming that social encounters must start with the attempt to recognize the intentions of the other actor (Baggs, 2020; Gallagher, 2020).

## Extended Skill Learning and Ecological–Enactive Cognitive Science

We began this paper by noting a tension that exists in how the notion of *skill* is understood in current theoretical work in ecological and enactive approaches in cognitive science. Skill is understood ambiguously as either a property of the animal’s body or as property of the extended animal–environment system. Our aim has been to resolve this tension by rejecting the first understanding in favor of the second. We have drawn on work from the empirical literature that shows how skill learning is an inherently extended phenomenon. We suggest that adopting such a view of skill learning offers the most promising strategy for bringing the two theoretical approaches—ecological and enactive—together into an empirically productive synthesis. In this last section, we will briefly sketch some reasons for pursuing such a synthesis.

Historically, the fundamental difference between the two approaches has been in where they locate meaning (Baggs and Chemero, 2018). Ecological psychologists, following Gibson (1966, 1979), generally hold that meaning is external to the observer. The concept of affordances, in Gibson’s formulation, locates meaning in the environment (though note that this does not entail that meaning is independent of the features or the activities of the organism; see Segundo-Ortin et al., 2019). This theoretical move led to a productive empirical program. Rather than worrying about what is going on inside the organism, ecological psychologists are free to investigate the animal–environment relation by identifying repeatable structures and activities that occur in everyday life and seeking to understand the dynamics at play within those activities. The essence of the ecological empirical strategy is to study a highly constrained *task*, i.e., a repeated pattern of behavior that can be characterized in precise mathematical terms—things like steering a vehicle around an obstacle or bouncing a ball on a bat (see Warren, 2006). Ecological explanation, in short, is *task-oriented*.

Enactivists, meanwhile, are suspicious of task-oriented explanations. More precisely, enactivists feel that task-oriented explanations only capture behavior in an incomplete manner. Their concern is that such explanations seem to deny the agency of the actor. Enactivists prefer to think of meaning as an achievement of the actor (Varela et al., 1991, Thompson, 2007, Thompson and Stapleton, 2009). Enactivists seek an explanation of why a particular goal-directed activity comes about in the first place. As Di Paolo et al. (2017, p. 27) put it, enactivism “is concerned with explaining precisely [the] critical transitions between particular conditions that sometimes afford different functional descriptions and those ‘in-between’ dynamics that (re)constitute these or novel conditions” (for a more detailed discussion of this difference in explanatory strategy, see Baggs, 2018).

The approach to skill learning that we have been outlining in this paper is undeniably in the task-oriented tradition, in the sense just identified. We have suggested that skills should be understood in terms of enabling constraints, but enabling constraints can be understood as constraints only relative to a goal. By invoking enabling constraints, we are already presupposing that the actor is engaging in some goal-directed activity, for example, that the actor is already *trying* to stand upright or to get around her environment. We are not explaining, as the enactivists wish to explain, *why* it is that this particular actor is even trying to stand upright right now, in this particular context—i.e., why did this goal arise in the first place?

Researchers within the ecological and enactive approaches, it seems, are pursuing two quite different projects. Is this a fatal problem for the prospect of an enactive–ecological rapprochement? Possibly. But we would like to suggest that it need not be. The key here is that the task-oriented mode of explanation in ecological psychology can be interpreted as a methodological strategy rather than as an ontological framework. To talk of tasks and constraints is not necessarily to reify those tasks and constraints (that is, it need not be the case that the actor herself sees the world in terms of tasks). Rather, a task-oriented approach can be seen as merely a useful methodological tool for empirically getting to grips with at least some subset of the behaviors that actors engage in (specifically, it allows us to empirically investigate just those activities that are susceptible to a characterization in terms of optimization relative to some perceptual variable. Activities that cannot be so characterized fall outside the scope of present-day ecological explanation).

Enactivists have long emphasized the need to understand the animal system in terms of its developmental history. A central notion in enactivism is that of *structural coupling*, which was defined by Maturana and Varela (1987, p. 75) as follows: “We speak of structural coupling whenever there is a history of recurrent interactions leading to the structural congruence between two (or more) systems.” An example of such a history of interaction is the co-evolution of automobiles and cities: for example, the more people rely on cars to get around, the more the city develops on the model of urban sprawl (Maturana and Varela 1987, p. 99). In terms of the learning organism, change over time is understood as a “structural drift” (bodily change) that occurs as the organism continually maintains the conditions for its own viability (Maturana and Varela 1987, p. 170).

This understanding of learning has been substantially developed by Di Paolo et al. (2017, p. 152), who (as mentioned earlier) propose that learning should be understood as the construction and maintenance of a network of sensorimotor schemes. These schemes should be understood, they note, not as something that is confined to the body of the individual actor (like a set of motor algorithms), but as spanning the animal–environment system: “it is important to stress that sensorimotor schemes, and networks of these, constitutively involve both the organic body and its environment.” But, again, the maintenance of the network is understood in terms of the organism’s maintaining the conditions for its own viability. Specifically, Di Paolo and colleagues propose to adopt a version of Piaget’s theory of equilibration, which conceives the learner as repeatedly attaining new stable forms of organization by repeatedly engaging with the environment (Di Paolo et al., 2017, p. 85).

One way to contrast the way that learning is understood in the ecological and enactive approaches is to say that for enactivists, learning is understood as a process of construction and self-maintenance, while for ecological psychologists learning is a process of attunement. On the enactivist perspective, the things the animal learns to do are constructed by the animal. In Maturana and Varela’s early work, this construction process has no inherent direction, but is understood simply a process of “structural drift.” In the formulation of Di Paolo et al. (2017) there is a direction to the process and the direction arises from a dialectical confrontation between newly encountered worldly structures and the organism’s existing structure: equilibration is what happens when the organism successfully re-organizes itself so as to incorporate an appropriate response to the newly encountered structure. For ecological psychologists, by contrast, learning cannot be understood as a process of construction at all. Learning must be understood instead as being directed toward specific structures that already exist in the environment. This is most clear in Jacobs and Michaels (2007) proposal that there exists “information for learning,” i.e., information that is available in ambient energy which the learner is in principle able to detect. By detecting this information, the learner discovers in which direction to adjust its activity in order to optimize its performance relative to some task goal (see also Raja, 2019, p. 337). On this account, then, the “end point” of learning already exists at the beginning of the process, in a sense.

On the face of it, it would appear that a comprehensive embodied theory of learning will need to synthesize both of these perspectives. To appeal only to a process of individual equilibration or sense-making seems insufficient: if learning is all just about incorporating novel structures into our body schema, then why is it then we end up behaving in such remarkably similar ways to one another? Why, for instance, do we end up speaking basically the same language as those around us? Or consider the question of why infants bother to transition from crawling to walking (Adolph et al., 2012). Why do not some of us simply carry on crawling? The direction that exists within learning seems to come, in at least some sense, from the learner’s seeking out of more effective ways of doing things that already exist as possibilities in the environment: walking would seem to already exist as a possibility that the toddler can strive toward, and not merely as a perturbation that has to be incorporated into the existing system. On the other hand, if we can only learn to perform actions that already exist as possibilities in the structure of the environment in some sense, then how do we ever come to do anything inventive, like coming up with new cooking recipes or telling jokes?

Once we understand skill learning as an extended phenomenon, as we have advocated above, it becomes possible to see how an ecological–enactive synthesis might be pursued. What is needed is an account that recognizes *both* the autonomously generated exploratory behavior of the organism *and* the pre-existing structure of the environment toward which that exploratory behavior is directed.

A view of the learner’s exploratory behavior as being directed at a structured environment is already central to Eleanor J. Gibson’s ecological approach to learning and development (Gibson, 1988; Adolph, 2019). This is well expressed in a paragraph from a recent paper (Adolph, 2019):

“Eleanor Gibson … said that watching children on a playground is a revelation of attention to affordances. Children swoosh down, climb up, and hide under the chute of the slide. They swing on the monkey bars, hang by their knees, and balance upright on the rungs. Any small object presents a compelling opportunity for infant exploration with hands, eyes, and mouth. Infants carry objects to share with their caregivers, to place in different locations, and for no discernible reason except their apparent delight in carrying things that afford carrying… Even in a seemingly empty room, infants find things to do. They poke their fingers into indents in the floor, pick up tiny crumbs from the carpet, and use any small protuberance to try to climb the walls.”

An extended account of skill learning must begin with an understanding of exploratory behavior and play as situated in an environment that already has structure.

Ultimately, the reason that it is important to clarify what we mean by skills is that the notion of skill is central to an ecological-enactive theory of learning. If we are going to give skills a central position in our theorizing, then we ought to develop an explicit theory of learning too. The notion of enabling constraints can potentially provide a valuable tool in this project. For historical reasons, learning has been marginalized in embodied theory. It is time to put learning back into the heart of things. Giving learning a central position in radical embodied cognitive science is, we contend, the most promising strategy for unifying the ecological and enactive approaches. The payoff of this theoretical effort is potentially a much more powerful approach to embodied cognitive science in general.

## Author Contributions

All authors listed have made a substantial, direct and intellectual contribution to the work, and approved it for publication.

### Conflict of Interest

The authors declare that the research was conducted in the absence of any commercial or financial relationships that could be construed as a potential conflict of interest.
